# The evolution of metabolic networks of *E. coli*

**DOI:** 10.1186/1752-0509-5-182

**Published:** 2011-11-01

**Authors:** David J Baumler, Roman G Peplinski, Jennifer L Reed, Jeremy D Glasner, Nicole T Perna

**Affiliations:** 1 Genome Center of Wisconsin, University of Wisconsin-Madison, Madison, Wisconsin, USA; 2 Department of Chemical and Biological Engineering, University of Wisconsin-Madison, USA; 3 Department of Genetics, University of Wisconsin-Madison, USA

## Abstract

**Background:**

Despite the availability of numerous complete genome sequences from *E. coli *strains, published genome-scale metabolic models exist only for two commensal *E. coli *strains. These models have proven useful for many applications, such as engineering strains for desired product formation, and we sought to explore how constructing and evaluating additional metabolic models for *E. coli *strains could enhance these efforts.

**Results:**

We used the genomic information from 16 *E. coli *strains to generate an *E. coli *pangenome metabolic network by evaluating their collective 76,990 ORFs. Each of these ORFs was assigned to one of 17,647 ortholog groups including ORFs associated with reactions in the most recent metabolic model for *E. coli *K-12. For orthologous groups that contain an ORF already represented in the MG1655 model, the gene to protein to reaction associations represented in this model could then be easily propagated to other *E. coli *strain models. All remaining orthologous groups were evaluated to see if new metabolic reactions could be added to generate a pangenome-scale metabolic model (iEco1712_pan). The pangenome model included reactions from a metabolic model update for *E. coli *K-12 MG1655 (iEco1339_MG1655) and enabled development of five additional strain-specific genome-scale metabolic models. These additional models include a second K-12 strain (iEco1335_W3110) and four pathogenic strains (two enterohemorrhagic *E. coli *O157:H7 and two uropathogens). When compared to the *E. coli *K-12 models, the metabolic models for the enterohemorrhagic (iEco1344_EDL933 and iEco1345_Sakai) and uropathogenic strains (iEco1288_CFT073 and iEco1301_UTI89) contained numerous lineage-specific gene and reaction differences. All six *E. coli *models were evaluated by comparing model predictions to carbon source utilization measurements under aerobic and anaerobic conditions, and to batch growth profiles in minimal media with 0.2% (w/v) glucose. An ancestral genome-scale metabolic model based on conserved ortholog groups in all 16 *E. coli *genomes was also constructed, reflecting the conserved ancestral core of *E. coli *metabolism (iEco1053_core). Comparative analysis of all six strain-specific *E. coli *models revealed that some of the pathogenic *E. coli *strains possess reactions in their metabolic networks enabling higher biomass yields on glucose. Finally the lineage-specific metabolic traits were compared to the ancestral core model predictions to derive new insight into the evolution of metabolism within this species.

**Conclusion:**

Our findings demonstrate that a pangenome-scale metabolic model can be used to rapidly construct additional *E. coli *strain-specific models, and that quantitative models of different strains of *E. coli *can accurately predict strain-specific phenotypes. Such pangenome and strain-specific models can be further used to engineer metabolic phenotypes of interest, such as designing new industrial *E. coli *strains.

## Background

The gram-negative bacterium *E. coli *is one of the best-studied microorganisms. This bacterial species includes pathogenic strains that cause disease in various tissues in mammalian and other vertebrate hosts. Some of the more common diseases associated with pathogenic *E. coli *strains are caused by bacteria found in the gastrointestinal tract or urinary tract, and is a major cause of human morbidity and mortality worldwide. *E. coli *infections cost the healthcare industry over a billion dollars annually with the enterohemorrhagic (EHEC) and uropathogenic (UPEC) *E. coli *strains alone responsible for more than 73,000 and 7,000,000 illnesses annually in the United States, respectively [1-3]. A number of genome sequences for these pathovars exist, and comparative analysis between commensal and pathogenic strains has revealed different virulence strategies [4-10]. However, the metabolic properties that differentiate these strains have not been thoroughly investigated. The metabolic content of the genomes of these strains is complex with each strain predicted to contain over 1,000 genes encoding metabolic enzymes and transporters [11]. One method to investigate the complexity of genome-scale metabolic networks is through the construction of computational models.

Computational modeling of bacterial metabolism offers a promising approach to predict strain-to-strain variation in metabolic capabilities and microbial strategies used during host association. The number of available genome-scale metabolic models (GEMs) has grown recently, and they capture the metabolic capabilities of numerous microbial taxa important to human health, biotechnology and bioengineering [12,13]. Systems biology combines computational and experimental approaches to study the complexity of biological networks at a systems level, where the cellular components and their interactions lead to complex cellular behaviors. Genome-scale biological networks have proven useful for interpreting high-throughput data and generating computational models. Mathematical models are constructed from network reconstructions, and they include variables, parameters, and equations to describe the potential behavior of these networks. Numerous types of genome-scale biological networks have been constructed including metabolic, regulatory, and transcriptional and translational machinery for *E. coli *K-12 [14-17].

To date, GEMs have been constructed for only two commensal strains of *E. coli*, *E. coli *K-12 (strain MG1655) and *E. coli *W [15,18]. The *E. coli *K-12 GEM has been used to engineer strains to increase valuable product formation [19-23], facilitate enzyme function discoveries [24], provide insight into the genome evolution of other enterobacteria [25,26], and improve the understanding of the connectivity of metabolic reactions within the cell [27]. Furthermore, computational metabolic models can be validated and refined by comparing *in silico *predictions with experimental data, where the discovery of disagreements or incorrect *in silico *predictions can lead to improvements and/or hypotheses about component interactions and unknown network components. An iterative process thus develops where the models are used to analyze experimental data and discrepancies lead to improved models and additional biological discovery. Such approaches have proven successful for updates to the *E. coli *models for regulation and metabolism [14,24,28,29].

Currently the construction of metabolic networks relies primarily on information derived from genome annotations, enzymatic/pathway databases, and published literature. By combining these resources, the elementally- and charged-balanced reactions catalyzed by enzymes associated with a given gene can be identified [30,31]. These reactions incorporate pertinent information such co-factors, substrates, products, reversibility, stoichiometry, and subcellular location. A genome-scale metabolic network contains a list of reactions, as well as the gene to protein to reaction (GPR) associations, and is used to formulate constraint-based GEMs. By comparing GEMs for pathogenic and non-pathogenic *E. coli *strains, metabolic differences can be identified that may lead to the development of new control strategies for *E. coli *associated disease.

Here we describe the construction of a detailed GEM for the pangenome of the species *E. coli*, and the use of this GEM to rapidly generate six strain-specific GEMs to compare genome-scale metabolism between four strains from two pathogenic lineages with two commensal K-12 strains. In addition, an ancestral *E. coli *core GEM was constructed consisting of only those metabolic reactions associated with genes that are conserved across 16 *E. coli *genomes. The metabolic potential of this ancestral core was also examined. Experiments were performed to iteratively refine and validate the six strain-specific GEMs under aerobic and anaerobic conditions. Once strain-specific GEMs were validated, the properties and metabolic differences distinguishing these pathogenic and commensal *E. coli *strains were computationally investigated, revealing that some pathogenic *E. coli *strains are more metabolically efficient than other strains in some environmental conditions. The *E. coli *GEMs generated in this work provide new tools for investigating the evolutionary and metabolic differences of these strains in conditions reflecting those environments encountered in human hosts. This is the first study to examine the metabolic properties of numerous strains of such a phylogenetically related group of microorganisms, and provides insight into the evolution of metabolism for the species *E. coli*.

## Methods

### Bacterial strains and growth conditions

Six *E. coli *strains and one *Salmonella *strain were used in this study (listed in Table 1). Frozen cultures were streaked onto Luria Bertani (LB) agar plates and grown overnight at 37°C. Isolated colonies were then used to inoculate MOPS (morpholinepropanesulfonic acid) minimal media (TekNova, Hollister, CA) and incubated overnight with shaking (220 rpm) at 37°C, and then overnight cultures were used to inoculate batch cultures grown with continuous sparging aerobically (70% N_2_, 25% O_2_, and 5% CO_2_) or anaerobically (95% N_2 _and 5% CO_2_) as previously described [32]. For carbon plate utilization assays, isolated colonies were used to inoculate Sheep Blood Agar plates (Biolog, Hayward, CA) and incubated at 37°C overnight aerobically or anaerobically in sealed Whirl-Pak^® ^Long-Term Sample Retention Bags (Nasco, Fort Atkinson, Wisconsin) saturated with an anaerobic gas mixture (95% N_2 _and 5% CO_2_) as described [33,34]. Anaerobic conditions were confirmed using an obligate aerobic bacterium that exhibited no growth and no respiration in any of the anaerobic conditions examined. Cells were collected and used to inoculate Biolog PM1 plates following the manufacturers recommendations with a minor modification of adding a top layer of mineral oil to each well for anaerobic culture conditions.

**Table 1:** List of bacterial strains used in this study.

Strain	Genotype	Source or reference
*E. coli *K-12 MG1655	Wild type	Dr. Patricia J. Kiley, University of Wisconsin-Madison [81]

*E. coli *K-12 W3110	Wild type	ATCC 39936

*E. coli *O157:H7 EDL933 (EHEC^a^)	Wild type	Dr. Charles W. Kaspar, University of Wisconsin-Madison [7]

*E. coli *O157:H7 RIMD/Sakai (EHEC)	Wild type	ATCC BAA-460 [6]

*E. coli *CFT073 (UPEC^b^)	Wild type	Dr. Rodney A. Welch, University of Wisconsin-Madison [10]

*E. coli *UTI89 (UPEC)	Wild type	Dr. Scott J. Hultgren, Washington University, St. Louis [5]

*Salmonella enterica *serovar *typhimurium *LT2	Wild type	Dr. Diana M. Downs, University of Wisconsin-Madison [82]

^a^Enterohemorrhagic *E. coli *(EHEC)

^b^Uropathogenic *E. coli *(UPEC)

### Updates to the E. coli K-12 MG1655 metabolic network

Prior to generating a pangenome GEM, additional genes in the genome of *E. coli *K-12 MG1655 were evaluated as possible updates to the most recent *E. coli *GEM (iAF1260)[15]. The annotations for *E. coli *K-12 MG1655 were obtained and examined from the ASAP, EcoGene, KEGG, and EcoCyc databases [35-38]. ORFs encoding enzymes that were not included in iAF1260 were further investigated to develop elementally and charge-balanced reactions and to assign the reaction to a subcellular location based on pSORT predictions [39]. In some instances new ORFs were added as isozymes to existing reactions and the gene-to-protein-to-reaction associations updated. This resulted in the addition of 79 new ORFs to the iAF1260 GEM to create iEco1339_MG1655 (Additional file 1). Of the new 79 ORFs (Additional file 2), 62% were based on experimental data from the literature for *E. coli *strains [40-68] and the rest were based on sequence homology to enzymes already existing in *E. coli *metabolic networks or to experimentally characterized enzymes from other enterobacteria. These gene additions resulted in 42 new reactions, 32 new isozymes, and 30 new metabolites to the *in silico *model for *E. coli *K-12 MG1655.

### Generation of an E. coli pangenome metabolic network

Draft and complete enterobacterial genomes in the ASAP database have been continually updated using new publicly accessible genomes since the database's inception [35]. There are more than 150 genomes of enterobacteria in the ASAP database (along with predicted orthologs), 39 of which are *E. coli *genomes. Of these *E. coli *genomes, 16 are completely finished, and we have used the information from these genomes and that of *Salmonella typhimurium *LT2 (Table 2) to generate an *E. coli *pangenome metabolic network based on metabolic enzymes present in the union of 76,990 ORFs. Each ORF was assigned in the ASAP database to an ortholog cluster group (OCG), and the 76,990 ORFs map to 17,647 OCGs. This reduced the number of genes that had to be evaluated for inclusion in the metabolic network and allowed generation of strain-specific GPRs to rapidly be formulated (Additional file 3). Not all of these OCGs play a metabolic role and/or have sufficient experimentally determined details for metabolic network inclusion. For example only 32.3% of ORFs in the total genome (1,339/4,141) are accounted for in the updated GEM for *E. coli *K-12 MG1655 (iEco1339_MG1655). All of the gene to protein to reaction association information from the *E. coli *K-12 MG1655 (iEco1339_MG1655) and the *Salmonella *LT2 (iRR1083)[69] GEMs were mapped to the OCGs that contained the respective ORFs. The annotations for the genes composing the remaining OCGs were analyzed for additional new metabolic reactions and isozyme additions (Additional file 4) leading to the generation of an *E. coli *pan-GEM named iEco1712_pan (Additional file 5). All eight SBML files generated in this work were checked for syntax and internal consistency using the validation tool (http://sbml.org/validator/validate.php) and found to conform to all specifications of SBML through Level 3 Version 1 Core (Release 1).

**Table 2:** *E. coli *genomes used to construct the pangenome metabolic network.

Strain	ORFs	Genome number
*E. coli *K-12 MG1655	4,141	1

*E. coli *EDL933 (EHEC)^a^	5,196	2

*E. coli *53638 (EIEC)^b^	5,172	3

*E. coli *CFT073 (UPEC)^c^	4,889	4

*E. coli *E2348/69 (EPEC)^d^	4,652	5

*E. coli *EC4115 (EHEC)^a^	5,467	6

*E. coli *UTI89 (UPEC)^c^	4,944	7

*E. coli *E24377A (ETEC)^e^	4,953	8

*E. coli *Sakai (EHEC)^a^	5,253	9

*E. coli *SE11	4,973	10

*E. coli *APEC O1 (APEC)^f^	5,045	11

*E. coli *SMS-3-5	4,906	12

*E. coli *536 (UPEC)^b^	4,599	13

*E. coli *HS	4,393	14

*E. coli *ATCC 8739	4,236	15

*E. coli *K-12 W3110	4,171	16

*Salmonella enterica typhimurium *LT2^g^	4,506	-

^a^Enterohemorrhagic *E. coli *(EHEC)

^b^Enteroinvasive *E. coli *(EIEC)

^c^Uropathogenic *E. coli *(UPEC)

^d^Enteropathogenic *E. coli *(EPEC)

^e^Enterotoxigenic *E. coli *(ETEC)

^f^Avian pathogenic *E. coli *(APEC)

^g^Included for comparative purposes

### Flux Balance Analysis

Fluxes through metabolic network reactions can be predicted using flux balance analysis (FBA) [70]. In FBA, fluxes are constrained by steady-state mass balances, enzyme capacities and reaction directionality. These constraints yield a solution space of possible flux values, and FBA uses an objective function to identify flux distributions that maximize (or minimize) the physiologically relevant predicted solution. Cellular growth rate (or biomass production) is often used as an objective function for FBA [71], and was used for FBA analyses performed in this study. The same biomass equation, growth (GAM) and non-growth (NGAM) associated ATP requirement values, and PO (number of ATP molecules produced per pair of electrons donated to the electron transport system) ratio were used for all *E. coli *developed models, and were the same as that in iAF1260[15]. For FBA and dynamic simulations the reported [15] wildtype biomass was used and for determination of essential reactions the core biomass was used. Using FBA, *in silico *predictions of growth yield, growth rate, and carbon source utilization were compared to experimentally determined values for all six *E. coli *strains and for *Salmonella *LT2 in both aerobic and anaerobic conditions (Additional files 6 and 7). For carbon source utilization and gene deletion simulations, a maximum uptake rate of 10 mmol per gram of dry weight per hour (mmol/gDW cell/h) was used. FBA was also used to predict essential reactions by constraining reactions to have zero flux and maximizing growth rate. If the resulting maximum predicted growth rate (using FBA) was zero then the reaction was considered to be essential. Reaction deletion simulations were evaluated under both aerobic and anaerobic conditions.

### Batch Growth Experiments and Simulations

We performed dynamic FBA simulations of batch growth [72] and compared these results with experimental data (Additional file 6). In the laboratory, cells were grown overnight in MOPS minimal medium with the addition of glucose (11 mM) as the sole carbon source, and used to inoculate batch cultures to an optical density (OD_600 nm_) of ~0.02. Batch growth under aerobic and anaerobic conditions was conducted at 37°C and spectrophotometric measurements, viable cells/ml, and biomass (g dry cell weight) were determined at each time point. Samples were collected every hour and passed through a 0.2 μm syringe filter, and then frozen at -70°C for subsequent UPLC analysis. The filtered supernatants were then analyzed to determine glucose concentrations at each time point using a UPLC following manufacturers recommendations (Waters Co., Milford, MA). Growth rates, growth yields, and glucose specific uptake rates were determined from experimental data using the linear least squares estimate as described [73]. Biomass to OD_600 _conversion values were also calculated for each strain (Table 3). These conversion values were used to estimate initial biomass (T_0_) values using the initial OD_600 _measurements (O.D.600 = ~0.02) for each experiment. The T_0 _values were used as parameters for dynamic FBA simulations of batch growth to determine the exponential growth rates (1/h), biomass yields (g biomass/1g glucose), and times (h) needed to reach stationary phase for the corresponding experimental conditions (aerobic or anaerobic growth in MOPS with 11 mM glucose). For each strain, experimental values from three biological replicates were then compared to those calculated from three computational simulations with matching starting biomass values under aerobic and anaerobic conditions.

**Table 3:** Experimental strain-specific conversion factors for aerobic or anaerobic growth conditions.

	Biomass (gDW/L) to OD_600 _± SE		Viable cells (CFU/ml) to OD_600 _± SE		Biomass (ng) to Viable cells (CFU)	
***E. coli *Strain**	**Aerobic**	**Anaerobic**	**Aerobic**	**Anaerobic**	**Aerobic**	**Anaerobic**

K-12 MG1655	0.415 ± 0.013	0.468 ± 0.015	1.56*10^9 ^± 1.04*10^8^	1.40*10^9 ^± 5.00*10^7^	0.266	0.334

K-12 W3110	0.410 ± 0.018	0.442 ± 0.020	8.86*10^8 ^± 4.94*10^7^	1.15*10^9 ^± 7.35*10^7^	0.463	0.384

EDL933	0.436 ± 0.018	0.543 ± 0.010	2.00*10^9 ^± 4.09*10^8^	3.00*10^9 ^± 1.20*10^8^	0.218	0.181

Sakai	0.376 ± 0.015	0.436 ± 0.007	9.20*10^8 ^± 9.91*10^7^	1.20*10^9 ^± 9.20*10^7^	0.409	0.363

CFT073	0.491 ± 0.019	0.525 ± 0.015	4.20*10^8 ^± 1.54*10^8^	2.10*10^9 ^± 1.60*10^8^	0.223	0.25

UTI89	0.380 ± 0.012	0.469 ± 0.015	1.00*10^9 ^± 6.80*10^7^	3.20*10^9 ^± 3.20*10^8^	0.38	0.147

*Salmonella *LT2	0.431 ± 0.010	0.459 ± 0.010	8.00*10^7 ^± 4.34*10^6^	5.00*10^8 ^± 3.84*10^7^	0.539	0.51

### UPLC analysis

Glucose analysis was conducted using an Acquity UPLC equipped with an Acquity BEH Glycan column (Waters). A mobile phase (75% [v/v] Acetonitrile/25% [v/v] H_2_O with 0.2% [v/v] Triethylamine; pH 9.1) was used at a flow rate of 0.1 ml/min to separate small molecules on a Waters Acquity UPLC equipped with an evaporative light scattering detector and photodiode array.

### Phylogenetic Analysis

A maximum parsimony phylogenetic analyses of seven taxa were conducted in MEGA4 [74] using a concatenated protein sequence data set of AcnA, GapA, IcdA, Mdh, MtlD, Pgi, and ProA with *S. typhimurium *LT2 used as the outgroup species. These genes were chosen since they have been successfully used for phylogenetic analyses of enterobacteria [75]. The alignment for this data set and subsequent maximum likelihood phylogenetic analyses was performed in MEGA4 using default parameters.

## Results

The metabolic model for *E. coli *K-12 MG1655 was developed 20 years ago and has undergone numerous improvements and updates. It is now a sophisticated compartmentalized GEM containing over 1,200 genes and 2,000 reactions. It has been used extensively for biotechnology and discovery applications. Here we generated a GEM for the pangenome of *E. coli*, and used the information from this larger metabolic network to generate strain-specific *E. coli *GEMs for two pathogenic lineages and an ancestral core GEM containing reactions conserved across all *E. coli *strains. Using this new collection of GEMs we validated strain-specific models by comparing predictions to experimental data, conducted a comparison of strain-specific GEMs from three *E. coli *lineages (commensal, EHEC, and UPEC), and examined the metabolic networks of numerous *E. coli *strains in an evolutionary perspective based on phenotypic traits.

### Updating the *E. coli *K-12 MG1655 metabolic model

**Figure 1:**
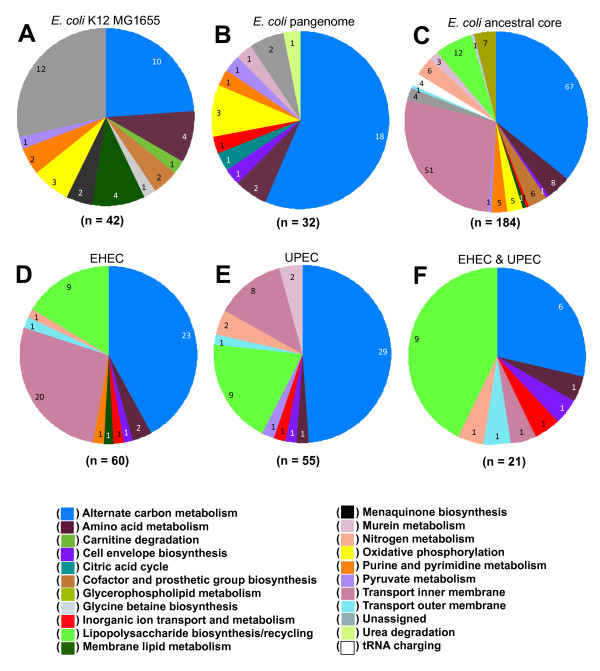
**A summary of metabolic reaction additions and deletions to GEMs used in this study**. In comparison to the previous *E. coli *K-12 MG1655 GEM (iAF1260), subsystem classification for new reaction additions to (A) iEco1339_MG1655. In addition, in comparison to iEco1339_MG1655, subsystem classification for reactions additions to (B) iEco1712_pan, or reaction deletions for (C) iEco1053_core, (D) reaction deletions shared in both EHEC strains (iEco1344_EDL933 and iEco1345_Sakai), (E) reaction deletions shared in both UPEC strains (iEco1288_CFT073 and iEco1301_UTI89), and (F) reaction deletions shared in both EHEC and EPEC strains.

The contents of the *E. coli *K-12 MG1655 genome were surveyed for new genes/reactions to add to the existing GEM (iAF1260). This effort added a total of 79 genes to iAF1260, of which 15 encoded proteins with significant similarity to proteins with characterized enzymatic activity, 15 were added based on orthology to genes found in the *S. typhimurium *LT2 GEM (iRR1083), and 49 were added based on experimental evidence from the scientific literature (Additional file 2). Three of the new genes were linked to metabolic reactions that were already included in iAF1260, but whose associated genes were previously unknown (i.e. orphan reactions). The 79 new genes added 42 new metabolic reactions and 30 new metabolites to the GEM. Exchange/transport reactions to permit cis-dihydrodiol-phenylacetyl-CoA utilization were also added resulting in an updated GEM for *E. coli *K-12 MG1655 designated as iEco1339_MG1655 composed of 1,339 genes, 1,069 metabolites, and 2,428 reactions (Additional file 1). This includes eight new reactions for phenylacetate metabolism that were added following our observation that *E. coli *K-12 MG1655 can grow in minimal media with phenylacetate as a sole carbon source (data not shown). The 42 new reactions in iEco1339_MG1655 were classified into 15 metabolic subsystems (Figure 1A). There were 24 genes added to the GEM that likely encode isozymes that participate in 32 existing reactions across 13 metabolic subsystems (Additional file 2). A total of 370 reactions in iEco1339_MG1655 contain multiple isozymes.

### Generation of an *E. coli *pangenome metabolic model

**Figure 2:**
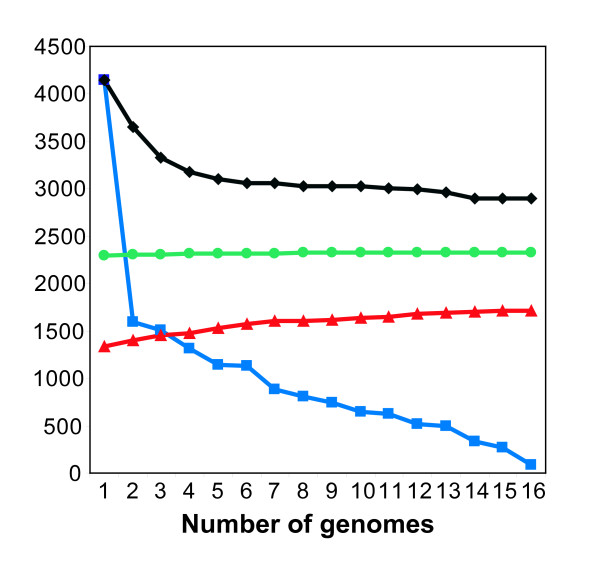
***Escherichia coli *core and pangenome metabolic network evolution according to the number of sequenced genomes listed in table 2**. Number of conserved genes (black diamonds), total number of unique genes without orthologs in prior genomes (blue squares), and total number of gene additions to the pan-GEM (red triangles), and total number of metabolic reactions additions to the pan-GEM (green circles) for a given number of genomes analyzed for the different strains of *E. coli*.

Understanding the evolution of metabolism for the species *E. coli *requires comparing genome-scale metabolic content among different strains of *E. coli *and its relatives. To faciltate these comparisons we mined the contents of 16 *E. coli *genomes to identify reactions that could be added to iEco1339_MG1655 to generate a pangenome metabolic network representing all metabolic reactions associated with genes present in any one of the *E. coli *genomes (Table 2). All genes from the sixteen *E. coli *and the *S. typhimurium *LT2 genomes (81,496 ORFs total) were classified into orthologous cluster groups (OCGs) based on ortholog relationships from the ASAP database [35]. This analysis resulted in a total of 17,647 OCGs with 16,417 representing the *E. coli *pangenome and 1,230 OCGs unique to *S. typhimurium *LT2. Of the 16,417 *E. coli *pangenome OCGs, 2,894 are found in all sixteen *E. coli *genomes, 4,146 are shared by two or more *E. coli *strains, and 9,377 are unique to individual *E. coli *genomes. Each additional genome added on average 806 new genes; however this number decreased as more genomes were analyzed (Figure 2).

A GEM for the pangenome was then constructed. Existing gene to protein to reaction (GPR) associations from GEMs of iEco1339_MG1655 and *S. typhimurium *LT2 (iRR1083) were mapped to their corresponding OCGs. The remaining OCGs were analyzed to see if they could be added to the pangenome metabolic network by adding additional isozymes or new reactions. This led to the addition of 373 OCGs and 32 new reactions beyond those found in the updated *E. coli *K-12 MG1655 GEM (iEco1339_MG1655), resulting in a pan-GEM, iEco1712_pan (Additional file 5), consisting of 1,712 genes, 1093 metabolites, and 2,452 reactions (Additional file 4). Each additional *E. coli *genome added to the pangenome metabolic analysis resulted on average added 27 new metabolic genes, 20 isozymes, and approximately 2 new metabolic reactions to the pan-GEM (Figure 2). The 32 reactions added to iEco1712_pan fall into 11 metabolic subsystems (Figure 1B), with the majority being related to alternate carbon metabolism (56%, associated with 4-hydroxyphenylacetate and propanediol metabolism) and oxidative phosphorylation (9%). Other added OCGs resulted in addition of isozymes associated with 14 metabolic subsystems, with the most abundant being alternate carbon metabolism (18%), cell envelope biosynthesis (11%), oxidative phosphorylation (11%), nitrogen metabolism (8%), glutamate metabolism (6%), and the remaining 9 subsystems consisted of a single reaction addition (Additional file 4).

### Generation of an *E. coli *core metabolic model

*E. coli *strains are thought to have diverged from a common ancestor ~10 million years ago (mya) [76] and it is of interest in understanding how strain-specific metabolism has evolved over time to have an estimate of the metabolic capabilities of an ancestral core for the species *E. coli*. We assume that genes conserved across the genomes of all strains represents a conservative estimate of the core genome of the ancestor of modern *E. coli *strains and used this collection of 2,894 conserved genes to construct an ancestral core GEM named iEco1053_core (Additional file 8). There are 1,053 of these genes in the *E. coli *K-12 MG1655 GEM (iEco1339_MG1655). The GEM for the *E. coli *ancestral core was made by removing OCGs and their associated reactions from the iEco1339_MG1655 GEM if one or more of the sixteen *E. coli *genomes did not have a gene assigned to the OCG (Additional file 3). If removing a reaction prevented biomass production for anaerobic growth on glucose (predicted using FBA) then the reaction was added back to the metabolic reconstruction without a gene associated with it and this occurred 24 times (Additional file 9). Using this approach 286 ORFS associated with 184 reactions and 177 isozymes were removed from iEco1339_MG1655 resulting in an *E. coli *ancestral core GEM (iEco1053_core) consisting of a total of 1,053 ORFs and 2,244 reactions (Table 4), and these 184 reactions we removed were classified based on metabolic subsystem (Figure 1C).

**Table 4:** *E. coli *strain-specific metabolic model information.

Strain	Additions			Deletions		Necessary reactions^a^	Total in model	
	
	ORFs	Reactions	Isozymes	ORFs	Reactions		ORFs	Reactions
K-12 MG1655	-	-	-	-	-	-	1,339	2,428

K-12 W3110	0	0	0	4	0	0	1,335	2,428

EDL933	38	8	20	51	60	10	1,344	2,376

Sakai	36	8	24	52	61	10	1,345	2,375

CFT073	9	2	25	85	66	9	1,288	2,362

UTI89	8	2	26	71	63	5	1,302	2,367

*E. coli *pangenome	79	32	255	-	-	-	1,712	2,452

*E. coli *core				286	184	24	1,053	2,244

^a^Necessary reactions without an associated gene for *in silico *models

### Characteristics of five new *E. coli *strain specific models

The pan-GEM was used to expedite the process of generating five new strain-specific *E. coli *GEMs, since the pangenome has reactions connected to cluster groups, a given strains genome contents were analyzed to identify what cluster groups its genes belong to and those associated reactions were included (Additional file 3). The *E. coli *strains we selected include an additional *E. coli *K-12 strain (W3110), two enterohemmoraghic *E. coli *O157:H7 strains (EDL933 and Sakai), and two uropathogenic strains (CFT073 and UTI89). Comparisons to iEco1339_MG1655, including the total number of strain-specific gene additions and deletions and the corresponding metabolic reactions are shown in Table 4. The two K-12 strains are laboratory strains derived from the same isolate and not surprisingly their GEMs, were very similar with the sole difference being removal of a few isozymes and a gene associated with galactitol transport from the W3110 GEM named iEco1335_W3110 (Additional file 10) due to a W3110-specific IS insertion in the *gatA *gene [77]. We built the five new *E. coli *GEMs named iEco1335_W3110 (Additional file 10), iEco1344_EDL933 (Additional file 11), iEco1345_Sakai (Additional file 12), iEco1288_CFT073 (Additional file 13), and iEco1301_UTI89 (Additional file 14) by deleting genes and reactions from the pan-GEM when missing from the genome under consideration (Additional file 15). If removing a reaction prevented biomass production for anaerobic growth on glucose (predicted using FBA) then the reaction was added back to the metabolic reconstruction without a gene associated with it. The number of these reactions without associated genes varied from 5 to 10 for each of the pathogenic *E. coli *strains (Table 4). Five of these reactions without associated genes were required in all four pathogenic *E. coli *GEMs (Additional file 9). Of the remaining five reactions, two were required for strains EDL933 and Sakai, one was required for strains EDL933, Sakai, and UTI89, and the remaining three are specific to *E. coli *strain CFT073.

Even though the genomes of the four pathogenic *E. coli *strains contain between 700-1,000 genes not present in the genome of *E. coli *K-12 MG1655, relatively few pathogen-specific metabolic genes were added to each GEM (Table 4). Eight new reaction additions were unique to the GEMs of the EHEC strains (iEco1344_EDL933 and iEco1345_Sakai) and consisted of urease, UDP-N-acetylglucosamine 4-epimerase, salicylate hydroxylase, gentisate 1,2,-dioxygenase, sucrose transport, tellurite reduction, fucose synthetase, and perosamine synthetase reactions. The two UPEC strain GEMs (iEco1288_CFT073, and iEco1301_UTI89) shared only one lineage-specific reaction addition for propionate CoA-transferase and each has a single strain-specific reaction addition unique to each strain; galactose isomerase activity for iEco1288_CFT073 and hydroxypyruvate reductase activity for iEco1301_UTI89.

**Figure 3:**
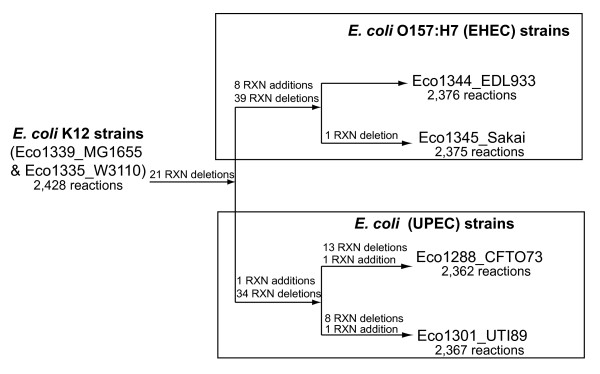
**A summary of lineage-specific reaction additions and deletions in comparison to the *E. coli *K-12 GEMs**.

In contrast to the relatively small number of gene and reaction additions there were a large number of reaction deletions for the pathogenic strain GEMs compared to iEco1339_MG1655 (Figure 3, Additional file 15). For EHEC strain GEMs, iEco1344_EDL933 and iEco1345_Sakai, there were 52 genes found in the *E. coli *K-12 MG1655 GEM (iEco1339_MG1655) that had no orthologous gene in the genomes of the two EHEC strains. These missing genes resulted in 60 reaction deletions in both iEco1344_EDL933 and iEco1345_Sakai, and these were classified based on metabolic subsystem (Figure 1D). There was only one additional reaction deletion unique to iEco1345_Sakai for D-cysteine desulfhydrase, whereas the ORF encoding this enzyme was still intact in the EDL933 strain. For each of the two UPEC strains there were 55 reactions that were missing in both iEco1288_CFT073 and iEco1301_UTI89 compared to iEco1339_MG1655, and these were further classified into metabolic subsystems (Figure 1E). Each of the two UPEC strains also contained numerous reaction deletions unique to each strain (Additional file 5).

When GEMs for all four pathogens (iEco1344_EDL933, iEco1345_Sakai, iEco1288_CFT073, and iEco1301_UTI89) were compared to those of the two K-12 strains (iEco1339_MG1655 and iEco1335_W3110), 21 shared reaction deletions were common to all four pathogenic *E. coli *strains, and they were categorized into the metabolic subsystems of alternate carbon metabolism, cell envelope biosynthesis, inorganic ion transport and metabolism, lipopolysaccharide biosynthesis, methionine metabolism, nitrogen metabolism, inner membrane transport, and outer membrane transport (Figure 1F).

### Assessment and validation of models for carbon source utilization

**Figure 4:**
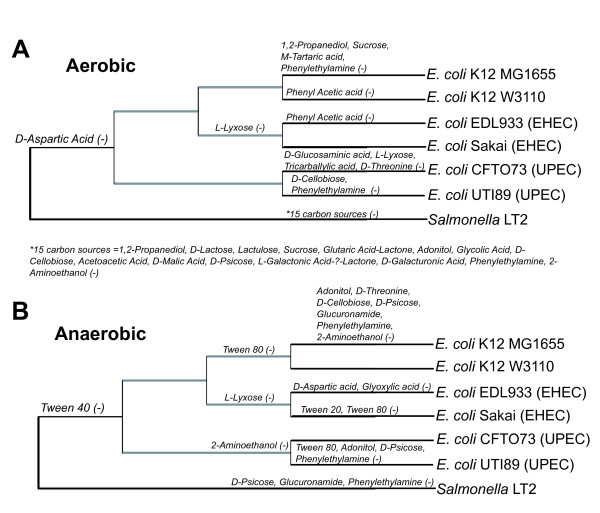
**Carbon source utilization results based on phylogeny of *E. coli *and *S. typhimurium *strains used in this study**. Experimental carbon source utilization results for both aerobic (A) and anaerobic conditions (B).

**Figure 5:**
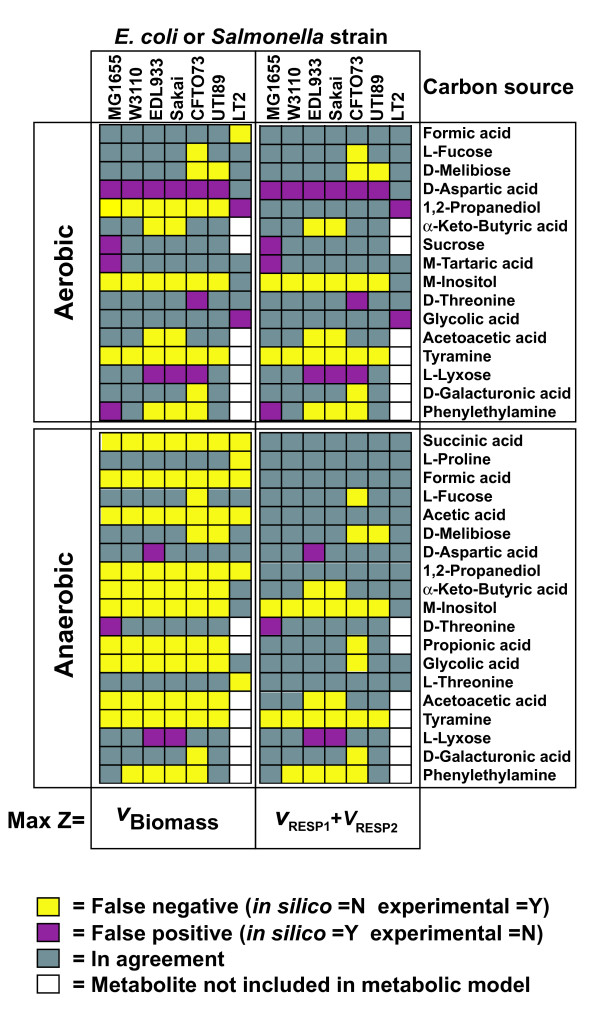
**Resolution of *in silico *and experimental carbon source discrepancies**. Carbon source utilization discrepancies for comparison of experimental and *in silico *data and the respective objective function (Z) used for flux balance analysis.

To evaluate the accuracy of the GEMs for all six *E. coli *strains, we examined each strain's ability to use different carbon sources under aerobic and anaerobic conditions using Biolog phenotypic arrays. There were numerous strain-specific differences in carbon source utilization in both aerobic (Figure 4A) and anaerobic conditions (Figure 4B). These experimental results were then compared to FBA predictions of growth using different carbon sources. For those compounds included in the Biolog plates that have transporters in the model, FBA was used to predict if they could be used for growth as sole carbon source. This included 76 potential carbon sources for the six *E. coli *strains and 54 potential carbon sources for *Salmonella *LT2. If FBA calculated a zero growth rate then the compound was predicted not to be usable as a sole carbon source, while positive calculated growth rates indicated that the model predicted the compound could be used as the sole carbon source. Of the 76 compounds, there were 59 (aerobic) and 56 (anaerobic) carbon sources where model predictions and experiments agreed for all six *E. coli *models (Additional file 7). The 16 and 19 carbon sources with discrepancies between *in silico *and experimental results in at least one model (shown in Figure 5) fall into two categories i) when strains did not grow and the model predicted growth (false positive), and ii) instances where strains grew and the model predicted no growth (false negative).

For aerobic carbon source utilization, the number of false positives varied from one to four, and the most accurate models (one false positive each) were for *E. coli *K-12 W3110 (iEco1335_W3110) and UTI89 (iEco1301_UTI89) (Figure 5). The number of aerobic false negatives was greater than false positives and ranged from three to seven with the least observed for iEco1339_MG1655 and the most observed for iEco1288_CFT073. Of these model-data discrepancies, there were some carbon sources that led to inaccurate predictions by all six *E. coli *strain specific models such as the utilization of D-aspartic acid (false positive) and M-inositol and tyramine (false negative). The two pathogenic lineages (EHEC or UPEC) exhibited some lineage-specific false negatives for alpha-keto-butyric acid and acetoacetic acid utilization (iEco1344_EDL933 and iEco1345_Sakai) or D-melibiose (iEco1288_CFT073 and iEco1301_UTI89). FBA predictions using a mixture of L- and R- isomers of 1,2 propanediol (a racemic mixture of 1,2 propanediol is used as the sole carbon source in Biolog PM1 plates) resolved aerobic false negative discrepancies for 1,2 propanediol for five *E. coli *strains, without introducing any new false positives (data not shown).

For anaerobic carbon source utilization, there were no false positives observed for three *E. coli *strains (iEco1335_W3110, iEco1288_CFT073, and iEco1301_UTI89) and either one or two false positives observed for carbon sources such as D-Threonine (iEco1339_MG1655), D-Aspartic acid (iEco1344_EDL933), and L-Lyxose (iEco1344_EDL933 and iEco1345_Sakai). In contrast to the aerobic results, there were generally more false negative than false positive predictions, with the number of false negatives ranging from 10 (for iEco1339_MG1655) to 14 (for iEco1288_CFT073) for the six *E. coli *strains (Figure 5). Of these compounds associated with anaerobic false negatives, there were 10 that led to inaccurate growth predictions for all six *E. coli *strains (succinic acid, formic acid, acetic acid, 1,2-propanediol, alpha-keto-butyric acid, M-inositol, propionic acid, glycolic acid, acetoacetic acid, and tyramine) (Figure 5).

The Biolog phenotype assay uses reduction of a colorimetric tetrazolium dye to measure microbial respiration. Our initial FBA predictions used an objective function that relates to the ability of the bacterium to convert a particular carbon source into biomass. There may be carbon compounds that the bacteria are able to metabolize but which do not result in measurable growth, thus leading to false negatives. To see if changing the objective function from biomass production to indicator dye reduction improves the FBA predictions under both aerobic and anaerobic conditions, two additional reactions were added to each of the models representing the movement of electrons from reduced quinones to the indicator dye used in Biolog plates (RESP1: mql8 => 2H^+ ^+ mqn8; and RESP2: q8h2 => 2H^+ ^+ q8). FBA was used again but a new objective function, equal to the sum of flux through these two new reactions, was maximized. If the maximum sum of fluxes was zero then the model predicted the carbon source could not be metabolized, while a positive sum of fluxes indicated a carbon source could be metabolized. These new FBA predictions (using respiration instead of growth as an objective) significantly reduced the number of anaerobic false negatives to between two (iEco1339_MG1655) and eight (iEco1288_CFT073) (Figure 5 and Table 5), while not affecting the number of false positives.

**Table 5:** Carbon source utilization with respiration as FBA objective function.

	*E. coli *K-12				*E. coli *O157:H7 (EHEC)^a^				*E. coli *(UPEC)^b^				*S. typhimurium *	
Strain	MG1655		W3110		EDL933		Sakai		CFT073		UTI89		LT2	

Condition	O_2_	No O_2_	O_2_	No O_2_	O_2_	No O_2_	O_2_	No O_2_	O_2_	No O_2_	O_2_	No O_2_	O_2_	No O_2_

Tested compounds included in models	76	76	76	76	76	76	76	76	76	76	76	76	54	54

True positive^c^	71	67	74	74	73	72	74	72	71	75	73	70	39	51

True negative^d^	5	9	2	2	3	4	2	4	5	1	3	6	15	3

False positive^e^	4	1	1	0	2	2	2	1	3	0	1	0	2	0

False negative^f^	3	2	2	3	5	5	5	5	6	8	3	3	0	0

^a^Enterohemorrhagic *E. coli *(EHEC)

^b^Uropathogenic *E. coli *(UPEC)

^c^Experimental = Y

^d^Experimental = N

^e^Experimental = N and *in silico *= Y

^f^Experimental = Y and *in silico *= N

Overall, once the FBA objective was changed from biomass to respiration, all *E. coli *models exhibited a statistically significant relationship between model predictions and experimental growth phenotypes (chi-squared test statistic yields p < 0.05) for both aerobic (>88% accurate) and anaerobic (>89% accurate) conditions (Table 5). The carbon sources M-Inositol and tyramine still led to false negative predictions for all *E. coli *models examined under both aerobic and anaerobic conditions, which may indicate that missing reactions or gaps may exist in pathways for utilization of these carbon compounds. When considering both the aerobic and anaerobic conditions the overall accuracy for individual strain-specific models was iEco1301_UTI89 (95.3%), iEco1335_W3110 (94.7%), iEco1339_MG1655 (93.4%), iEco1345_Sakai (91.4%), iEco1344_EDL933 (90.8%), and iEco1288_CFT073 (88.8%).

### Batch growth predictions

**Figure 6:**
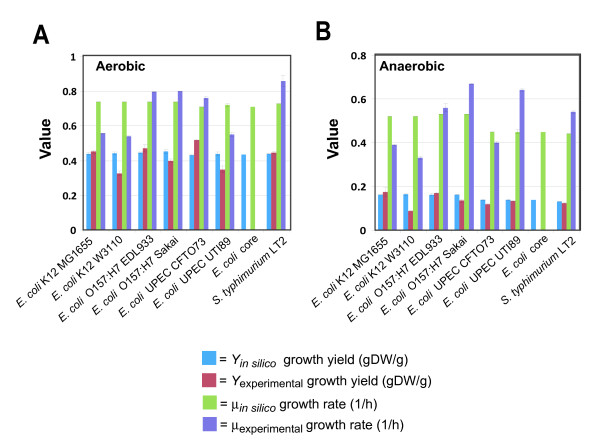
**Comparison of *in silico *and experimentally determined growth characteristics**. Strain specific batch growth values determined using MOPS minimal media with the addition of 0.2% glucose for (A) aerobic and (B) anaerobic growth conditions.

To further evaluate model predictions, dynamic FBA was used to predict time-courses (for substrate, product and cell concentrations), exponential growth rates (1/hr) and biomass yields (gDW cells/g glucose) for aerobic and anaerobic batch cultures in MOPS minimal media with the addition of glucose as the sole carbon and energy source. Batch culture experiments were conducted for each strain, and conversion factors for optical density to biomass, optical density to viable cell concentration, and biomass per viable cell values (Table 3) were determined for each strain in both anaerobic and aerobic conditions. These conversion values were then used to approximate starting biomass values used in the dynamic FBA simulations. For each *E. coli *strain, the maximum glucose uptake rates used for dynamic FBA were those reported for *E. coli *K-12 strain W3110 [73] (10 and 18.5 mmol glucose/gDW/h for aerobic and anaerobic conditions, respectively). The predicted growth rates and biomass yields from the model were then compared to experimental results of batch culture of each strain under anaerobic or aerobic conditions. For both aerobic or anaerobic growth conditions, the calculated growth rate (1/hr) for each *E. coli *strain was compared to the experimentally determined values (Table 6) and the agreement between *in silico *and experimental values was strong and significant (Pearson correlation test statistic yields *p *< 0.0002 for both aerobic and anaerobic conditions), yet when viewed separately for aerobic or anaerobic conditions, the correlation was not as strong and was not significant (Pearson correlation test statistic yields p < 0.37 for aerobic, and p < 0.45 for anaerobic conditions). In addition, the growth yields were calculated under aerobic or anaerobic growth conditions in MOPS minimal media for both aerobic and anaerobic growth conditions, and the *in silico *growth yields for each strain were compared to those determined experimentally (Figure 6) and the agreement between *in silico *and experimental values was strong and significant (Pearson correlation test statistic yields p < 0.0001 for both aerobic and anaerobic conditions).

**Table 6:** Comparison of experimental and *in silico *net specific growth rates (h^-1^).

	Aerobic			Anaerobic		
	
	**μ**_exp_	**μ***_In silico_*^a^	**μ***_In silicoII_*^b^	**μ**_exp_	**μ***_In silico_*^a^	**μ***_In silicoII_*^b^
*E. coli *MG1655	0.56 ± 0.03	0.74 ± 0.00	0.82 ± 0.05	0.39 ± 0.01	0.52 ± 0.00	0.19 ± 0.00

*E. coli *W3110	0.54 ± 0.01	0.74 ± 0.00	0.82 ± 0.00	0.33 ± 0.01	0.52 ± 0.00	0.19 ± 0.00

*E. coli *EDL933	0.79 ± 0.08	0.74 ± 0.00	0.63 ± 0.00	0.56 ± 0.04	0.53 ± 0.00	0.56 ± 0.01

*E. coli *Sakai	0.80 ± 0.01	0.74 ± 0.00	0.63 ± 0.00	0.68 ± 0.01	0.53 ± 0.00	0.56 ± 0.00

*E. coli *CFT073	0.76 ± 0.01	0.71 ± 0.00	0.60 ± 0.00	0.40 ± 0.01	0.45 ± 0.00	0.42 ± 0.00

*E. coli *UTI89	0.55 ± 0.02	0.72 ± 0.01	0.61 ± 0.01	0.64 ± 0.01	0.45 ± 0.01	0.42 ± 0.01

*E. coli *core	-	0.71	0.71	-	0.45	0.37

*E. coli *pangenome	-	0.74	0.73	-	0.53	0.42

*S. typhimurium *LT2	0.86 ± 0.05	0.73 ± 0.00	-	0.54 ± 0.01	0.44 ± 0.00	-

^a^Maximum oxygen uptake rates (15 mmol/gDW/h) and glucose uptake rates for aerobic (10 mmol/gDW/h) and anaerobic (18.5 mmol/gDW/h) conditions were used for *in silico *batch simulations in this work were those previously determined for *E. coli *W3110 from batch culture in M9 minimal media [73]

^b^Experimentally determined glucose uptake rate values from this work used for *in silico *batch simulations for *E. coli *K-12 (15.5 for aerobic and 8.1 for anaerobic), EHEC (7.9 for aerobic and 19.2 for anaerobic) or UPEC (7.7 for aerobic and 17.5 for anaerobic). For the core and pangenome models the average experimentally determined glucose uptake rate values from this work was used (10.3 for aerobic and 14.9 for anaerobic).

**Figure 7:**
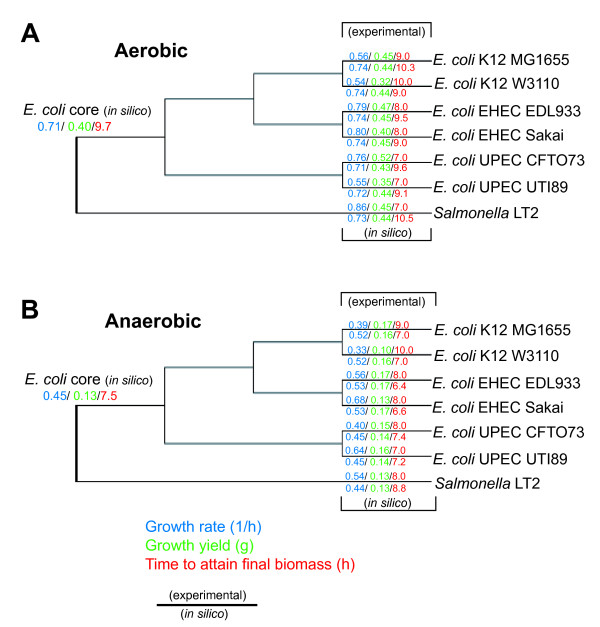
**Maximum likelihood phylogeny of *E. coli *and *S. typhimurium *strains used in this study constructed using the concatenated nucleotide sequence for 7 housekeeping genes**. *In silico E. coli *core and strain-specific experimentally determined growth rates (blue), growth yield (green), and time to attain final biomass during batch growth (red) during aerobic (A) or anaerobic conditions (B).

The maximum glucose uptake rate from one *E. coli *strain from each lineage K-12 (MG1655), EHEC (EDL933), UPEC (UTI89) was determined from experimental data for both aerobic and anaerobic conditions. These results revealed that the uptake rates for *E. coli *K-12 MG1655 were not similar to previously published values for *E. coli *K-12 W3110 (Table 6). When compared to the two pathogenic lineages, the two *E. coli *K-12 strains appear to have significantly higher glucose uptake rates in aerobic conditions but significantly lower glucose uptake rates in anaerobic conditions. The dynamic FBA simulations were repeated using the measured lineage-specific glucose uptake rates as parameters. The recalculated growth rates (Table 6), still showed a moderately strong correlation overall between *in silico *and experimental values (p < 0.0006), yet when viewed separately, resulted in a decreased correlation for aerobic conditions, and a increased correlation between *in silico *and experimental values for anaerobic conditions (Pearson correlation test statistic yields *p *< 0.00003). Figure 7 shows a phylogenetic tree of the strain relationships plotted along with the growth rate data, displaying the aerobic and anaerobic growth rates calculated without the uptake rate correction. Additionally we determined the amount of time needed to reach stationary phase experimentally and computationally using dynamic FBA for each strain in each condition. Evaluation of these results reveal that some of the pathogenic lineages attain final biomass in less time compared to the *E. coli *K-12 strains (Table 7).

**Table 7:** Time (h) to reach final biomass values in batch growth under both aerobic and anaerobic conditions.

Strain	Aerobic		Anaerobic	
	
	*In silico*	Experimental	*In silico*	Experimental
*E. coli *K-12 MG1655	10.3 ± 0.3	9.0 ± 0.0	7.0 ± 0.1	9.0 ± 0.0

*E. coli *K-12 W3110	9.0 ± 0.3	10.0 ± 0.0	7.0 ± 0.1	10.0 ± 0.0

*E. coli *EDL933	9.5 ± 0.1	8.0 ± 0.0	6.4 ± 0.1	8.0 ± 0.0

*E. coli *Sakai	9.0 ± 0.0	8.0 ± 0.0	6.6 ± 0.1	8.0 ± 0.0

*E. coli *CFT073	9.6 ± 0.1	7.0 ± 0.0	7.4 ± 0.1	8.0 ± 0.0

*E. coli *UTI89	9.1 ± 0.2	7.0 ± 0.0	7.2 ± 0.1	7.0 ± 0.0

*E. coli *core	9.7 ± 0.0	-	7.5 ± 0.0	-

*Salmonella *LT2	10.5 ± 0.0	7.0 ± 0.0	8.8 ± 0.2	8.0 ± 0.0

### Analysis of reaction essentiality

To further explore the metabolic differences and similarities between all six *E. coli *strain-specific GEMs, we compared reaction essentiality predictions for *in silico *conditions simulating aerobic and anaerobic growth in glucose minimal media. The number of predicted essential reactions shared in common for all six *E. coli *strains and also the *E. coli *ancestral core in both aerobic and anaerobic growth conditions was determined (n = 280) (additional file 16), and their corresponding reactions were further classified by metabolic subsystem (Table 8). Additionally for all six *E. coli *strains and the *E. coli *ancestral core, there were 15 additional conserved essential reactions predicted to be required under anaerobic conditions and these involve reactions assigned to subsystems for anaplerotic reactions (1), citric acid cycle (1), cofactor and prosthetic group biosynthesis (5), glycolysis/gluconeogenesis (2), purine and pyrimidine biosynthesis (1), inner membrane transport (2), and outer membrane transport (1). In addition to the shared predicted essential reactions for all *E. coli *strains examined, there were two lineage-specific reactions predicted as essential under both aerobic and anaerobic conditions for both EHEC strains (iEco1344_EDL933, iEco1345_Sakai), and the corresponding reactions were for fumarate reductase and glycolate oxidase. For the *E. coli *ancestral core (iEco1053_core), there were five additional reactions predicted as essential that were not predicted for any of the six *E. coli *strains (additional file 16), and these involve reactions assigned to subsystems for alanine and aspartate metabolism, glutamate metabolism, inner membrane transport, glycolate exchange, and outer membrane porin transport.

**Table 8:** Subsystem classification for essential reactions predicted for all six *E. coli *strains under aerobic conditions (n = 282).

Subsystem	Number of essential genes	Percentage(%)
Alternate Carbon Metabolism	3	1.1

Amino Acid Metabolism	151	53.5

Cell Envelope Biosynthesis	41	14.5

Citric Acid Cycle	4	1.4

Cofactor and Prosthetic Group Biosynthesis	72	25.5

Folate Metabolism	3	1.1

Glycerophospholipid Metabolism	12	4.3

Inorganic Ion Transport and Metabolism	7	2.5

Lipopolysaccharide Biosynthesis/Recycling	11	3.9

Membrane Lipid Metabolism	2	0.7

Murein Biosynthesis	2	0.7

Nucleotide Salvage Pathway	8	2.8

Purine and Pyrimidine Biosynthesis	19	6.7

Transport, Inner Membrane	3	1.1

Transport, Outer Membrane	13	4.7

Unassigned	1	0.4

## Discussion

This study describes the generation of GEMs representing the union (pangenome) and also the intersection (core) of all identifiable metabolic reactions contained in sixteen genomes of *E. coli*. We used the *E. coli *pan-GEM to rapidly construct six *E. coli *strain-specific GEMs. A comparison between model growth predictions and Biolog phenotypes measured in the laboratory demonstrated an accuracy of more than 88%, including those under anaerobic conditions Additional quantitative data was generated for each strain and used to validate the correlation between model predictions and experimental physiology of the strains in the laboratory. These new *E. coli *GEMs serve as a framework to examine genome-scale metabolic similarities and differences between strains in an evolutionary context with respect to the commensal, EHEC, and UPEC lineages.

The two *E. coli *K-12 strains (MG1655 and W3110) are widely used laboratory strains that are believed to have diverged from the same parental strain (strain EMG2 or WG1) approximately 50 years ago [77]. The sole identified metabolic differences between the two *E. coli *K-12 strains based on genome comparison include the *gatA *gene that is involved in galactitol transport, *dcuA *and *dcuC *involved in C4-dicarboxylate transport metabolism, and also *tnaB *thought to be involved in the utilization of tryptophan as a carbon and/or nitrogen source [77]. Of these four metabolic gene differences, only inactivation of *gatA *leads to a loss of a reaction in iEco1335_W3110, compared to iEco1339_MG1655 since *dcuA*, *dcuC *and *tnaB *have other isozymes. The *gatA *gene contains an insertion sequence (IS) element in *E. coli *W3110, which suggests a phenotypic loss for galactitol utilization as a carbon source, yet experimental data (Figure 5) reveals that the strain can still use this substrate as sole carbon source, indicating that other transporters may permit galactitol transport for *E. coli *W3110. Although the two *E. coli *K-12 strains (MG1655 and W3110) exhibited no differences in their GEMs, quantitative and strain-specific differences were observed during batch growth in minimal media with glucose as the sole carbon source. While *in silico *predictions for growth yield were similar for iEco1339_MG1655 and iEco1335_W3110, experimental data reveal that in both aerobic and anaerobic conditions, strain MG1655 had higher growth yields, higher growth rate, and attained the final biomass value in less time than strain W3110 (Figure 7). Therefore, although the *in silico *models for these two strains are nearly indistinguishable, strain specific differences in complex traits such as biomass composition [78], ATP requirements, PO ratios, and glucose uptake rates may account for these experimental differences. Previous studies have shown that despite their nearly identical genomes and very similar growth patterns in a bioreactor, W3110 and MG1655 have many significant differences in their transcriptomes and proteomes. These include differential expression of pathways affecting central metabolism and the generation of precursor metabolites and energy [79] suggesting that future models for even these very similar strains will need to account for subtle genetic differences between strains to accurately predict phenotypic traits in simulated culture conditions.

Previous analyses of the *E. coli *pangenome estimated that on average each new *E. coli *genome sequence added about 176 unique genes to the pangenome [8,9], and among these unique genes, we found each additional *E. coli *genome resulted in 27 metabolic gene additions corresponding to about 2 new metabolic reactions and 20 isozymes suitable for inclusion in the pan-GEM (Figure 2). Clearly some of the metabolic differences between *E. coli *strains are due to the addition of genes with new metabolic activity. However, our ability to add new reactions to the metabolic reconstructions is severely limited by the paucity of experimental characterization of the metabolic genes, proteins, and reactions unique to pathogenic strains. Since the strain-specific portions of the genomes remain largely uncharacterized, our current understanding of the metabolic functions they encode is dominated by the presence and absence of genes encoding functions represented in the iEco1339_MG1655 GEM. Many of the genes included in this model are not universally conserved among the genomes we examined; resulting in strain-specific GEMs with an average of 70 fewer genes than iEco1339_MG1655 (Table 9). This observation is also consistent with draft GEMs generated using the Model SEED [80] where the GEM for *E. coli *MG1655 contained more genes (>60) and reactions (>460) than the draft GEMs for all four pathogenic *E. coli *strains examined in this work (data not shown).

**Table 9:** Number of strain-specific orthologous genes in common with those contained in iEco1339_MG1655

Strain	ORFs
*E. coli *K-12 MG1655	1,339

*E. coli *EDL933 (EHEC)	1,260

*E. coli *53638 (EIEC)	1,226

*E. coli *CFT073 (UPEC)	1,234

*E. coli *E2348/69 (EPEC)	1,221

*E. coli *EC4115 (EHEC)	1,247

*E. coli *UTI89 (UPEC)	1,242

*E. coli *E24377A (ETEC)	1,292

*E. coli *Sakai (EHEC)	1,257

*E. coli *SE11	1,319

*E. coli *APEC O1 (APEC)	1,245

*E. coli *SMS-3-5	1,292

*E. coli *536 (UPEC)	1,229

*E. coli *HS	1,289

*E. coli *ATCC 8739	1,312

*E. coli *K-12 W3110	1,335

*E. coli *core	1,053

*Salmonella enterica typhimurium *LT2	1,135

Although carbon source utilization has become a standard method to assess the validity of computational metabolic model predictions, this study was the first to examine this procedure under anaerobic conditions. Initially, the accuracy of predictions for carbon source utilization during anaerobic conditions was less than those determined during aerobic conditions. We account this difference to comparisons between Biolog carbon source assays, which examine the ability of a microbial strain to generate energy from each sole carbon source, to *in silico *analysis that determines growth as a positive flux value for the biomass reaction. One possible explanation for experimental and *in silico *data discrepancies may be that a microbial strain may be able to generate energy from a given carbon source, but that the carbon source is not suitable to sustain growth (i.e. generate a positive biomass value). Therefore, rather than maximize the objective value for the biomass equation, we added two reactions to monitor the ability to generate energy through electron transfer to quinones, and in many cases this analysis resolved discrepancies between *in silico *predictions and experimental data, especially for anaerobic conditions. Although this methodology of examining carbon source utilization seems trivial, validation for accurate carbon source utilization is important for modeling complex environments such as those encountered in a host, as 31 of the 76 carbon sources tested here were used to simulate the conditions reflecting invasion of a human cell to study *S. typhimurium *LT2 infection [69]. Therefore, the validation of these strain-specific metabolic models for carbon source utilization will prove useful for future computational modeling of pathogenic *E. coli *strains in conditions encountered in the gastrointestinal tract or in other locations such as the urinary tract in mammalian hosts.

With the generation of the first GEMs for pathogenic *E. coli *strains, two EHEC strains and two UPEC strains, properties of these genome-scale metabolic networks were investigated to identify differences that may play a role in human disease. We analyzed two *E. coli *O157:H7 strains associated with foodborne outbreaks, strain EDL933 isolated from ground beef in the U.S in 1982 and strain Sakai isolated from contaminated radish sprouts that sickened thousands in Japan in 1996. Strains CFT073 and UTI89, which cause human disease outside of the intestine, were isolated from patients with acute urinary tract infections. A comparison of reaction deletions between the EHEC and UPEC metabolic networks reveals that the EHEC strains have more missing genes corresponding to reactions for inner membrane transport in comparison to the UPEC strains. In addition, the reaction deletions that occur in both pathogenic lineages relative to *E. coli *K-12 strains are mainly associated with genes involved in lipopolysaccharide biosynthesis/recycling and alternate carbon utilization. It seems likely that some of these missing reactions are the result of acquisition of genes during the evolution of the K-12 lineage. Perhaps some of the reactions missing from both pathogen lineages arise from parallel deletions arising from selective pressures common to both pathogens.

Batch growth experiments were conducted to compare growth yields, growth rates, and the amount of time to attain final biomass among strains. We were surprised that EDL933, Sakai and CFT073 have significantly higher growth rates than MG1655 during aerobic growth conditions yet the *in silico *predictions reveal little to no differences. We sought to determine if strain-specific glucose uptake rates may improve *in silico *growth rate predictions. Experimentally determined glucose uptake rates were actually lower for EDL933 and CFT073 than for MG1655, and did not improve *in silico *predictions. The growth yield values we measured in the laboratory also showed significantly (student's t-test statistic yields p < 0.05) higher yields for EDL933 and CFT073 than the two K-12 strains, but *in silico *predictions showed only minor strain-to-strain variations. Dynamic FBA using the strain specific *E. coli *GEMs predicts a similar growth rate from all models including the model for the ancestral core of *E. coli*. Yet the actual growth rates determined experimentally vary significantly between strains suggesting that our models are not accounting for some strain-specific factors such as oxygen uptake rates, biomass composition, ATP requirement parameters, or additional uncharacterized reactions. The length of time required to attain final biomass was significantly (student's t-test statistic yields p < 0.05) shorter for the four pathogens suggesting that they may be more efficient at biomass production during glucose catabolism, and dynamic FBA analysis accurately predicted this phenotypic difference among the strains.

In anaerobic batch growth conditions there were also differences between strains. All pathogenic strains have higher growth rates than the K-12 strains. The FBA predictions for EHEC strains both reflect this phenotype, but the *in silico *growth rate predictions for the UPEC strains did not reflect this trend. The experimentally determined glucose uptake rates are higher for both pathogenic lineages than K-12, and these organism-specific parameters improved the FBA predictions. The growth yields determined experimentally are significantly (student's t-test statistic yields p < 0.005) higher for the four pathogens than the K-12 strains. The length of time required to attain final biomass predicted by FBA and determined experimentally was significantly (student's t-test statistic yields p < 0.05) shorter for the EHEC strains than the K-12 strains. Overall, for anaerobic glucose catabolism, all four pathogens appear to grow better than both *E. coli *K-12 strains.

Even though the metabolic networks of each *E. coli *strain differ, there were relatively few strain-to-strain differences in reactions predicted as essential for the two growth conditions examined. While there were some identified for all strains that were unique for anaerobic growth in comparison to aerobic, there were relatively few differences between all strains. The two reactions (fumarate reductase and glycolate oxidase) predicted as essential for the *E. coli *O157:H7 strains, play essential metabolic roles for glycolate recycling and the reoxidation of menaquinol, and represent new targets for control strategies that may help to prevent and treat human EHEC illness.

The comparison of the pan- and core-GEMs reveals that a substantial fraction of the reactions in our current pan-GEM are also in the ancestral core-GEM (92%). However, our knowledge of the detailed biochemistry of the pangenome is likely incomplete since many of the genes in other *E. coli *strains have unknown functions. One reason why the number of reactions in the core- and pan-GEMs are so similar is because the genes that have been well-characterized biochemically in *E. coli *tend to be the genes that are conserved and likely ancestral. While the pathogenic *E. coli *strains are of great interest medically, they are not typically the focus of intense biochemical study to uncover the functions of their novel metabolic genes.

Overall, when data for aerobic conditions is viewed phylogenetically (Figure 7A), there is no clear trend specific to the two pathogenic lineages, yet it appears that *E. coli *CFT073 has evolved with a similar growth rate in comparison to the *E. coli *ancestral core predictions, where as all other strains have evolved with higher growth rates and yields (Figure 7A).

In contrast, in anaerobic conditions (Figure 7B), higher growth yields and faster batch growth performance were observed for both EHEC *E. coli *strains (EDL933 and Sakai), and the insight derived from *E. coli *ancestral core *in silico *predictions suggest that the UPEC and K-12 lineages have evolved with less efficient anaerobic glucose catabolism then the EHEC lineage. One possible explanation for this behavior may be that the K-12 and UPEC strains do not routinely encounter the selective pressure from anaerobic conditions, whereas the EHEC strains may have evolved for improved growth in anaerobic conditions enabling their growth in both bovine and mammalian GI tracts, thus suggesting that many EHEC strains may have a better-suited anaerobic metabolism for glucose utilization. These findings suggest that *E. coli *K-12 strains could be engineered to be more efficient for anaerobic batch growth and that other *E. coli *strains not examined in this work may yield similar results, yet additional studies are warranted to examine more *E. coli *strain-specific GEMs, quantitative parameters, and catabolism of additional substrates other than glucose.

## Conclusions

Here we have presented an update to the *E. coli *K-12 MG1655 GEM and an extensive new collection of GEMs for five *E. coli *strains including the first for two pathogenic lineages. These models have been validated through experimental data for aerobic and anaerobic conditions. This work demonstrated a new approach for validation of carbon source utilization, yielding accuracies of >88% for aerobic and anaerobic conditions for all six *E. coli *strains examined. In addition new lineage-specific quantitative data were generated and led to validation of the correlation between *in silico *predictions and experimental batch culture data for glucose catabolism during aerobic and anaerobic growth conditions. Thus, the iEco1339_MG1655, iEco1335_W3110, iEco1344_EDL933, iEco1345_Sakai, iEco1288_CFT073, and iEco1301_UTI89 GEMs provide new suitable platforms for computing cellular phenotypes in conditions reflecting those encountered in mammalian hosts such as the intestine or urinary tracts and for further integration of high throughput data generated from these bacterial strains during the course of infection in animal models.

Distinctive lineage-specific differences in the GEMs were identified and reveal that the main delineating metabolic factors between pathogenic and commensal *E. coli *strains are due to numerous gene/reaction deletions and not additions, and this observation was consistent with the number of genes and reactions contained in draft GEMs for all six *E. coli *strains generated using Model SEED. Historically many researchers have noticed that some pathogenic *E. coli *strains grow faster in comparison to commensal strains such as K-12, yet this phenotype has remained unexplained. These strain-specific models offer new tools for further investigation to determine precisely what combination of gene/reaction deletions account for the faster and more efficient biomass production observed experimentally for some of the pathogenic strains, thus providing new insight for bioengineering of industrial *E. coli *strains.

The generation of an *E. coli *pan-GEM (iEco1712_pan) consisting of all metabolic genes and reactions from 16 *E. coli *genomes, represents a new framework to rapidly generate additional *E. coli *strain/lineage-specific GEMs consisting of > 1,200 genes and >2,000 metabolic reactions. Finally, this study is the first to use a "paleo systems biology" approach to generate a GEM for an ancestral core of *E. coli *(iEco1053_core) providing the first insight to metabolic traits of an *E. coli *relative that may have existed ~10 mya, and demonstrated the use of an ancestral model to examine a closely related phylogenetic group of *E. coli *strains in the context of evolution.

## Authors' contributions

DB constructed the pangenome, core, and the six strain-specific GEMs and performed all *in silico *analyses. DB and RP obtained all of the experimental data. DB, JG, and NP designed the study. DB and JR analyzed and interpreted the data and performed the statistical analysis. DB, JG, JR, and NP wrote the manuscript. DB generated all eight SBML model files. All authors approve the content of this manuscript.

## Supplementary Material

Additional file 1 **Genome-scale metabolic model for *E. coli *K-12 strain MG1655**. SBML format of iEco1339_MG1655 for distribution and use in other modeling environments. Click here for file

Additional file 2 **Gene to protein to reaction (GPR) updates for *E. coli *K-12 MG1655 in the final version of the reconstruction**. This file contains two tables, the first contains all new GPR information added to the previous *E. coli *K-12 MG1655 GEM (iAF1260), and the second contains the final GPR information for iEco1339_MG1655. Click here for file

Additional file 3 **Orthologous gene cluster groupings for all ORFs in 16 *E. coli *genomes and one *Salmonella *genome**. This file contains the mapping of orthologous cluster group identifiers to each ASAP feature identifier and locus tag for all ORFs contained in the genomes of 16 strains of *E. coli *and one *Salmonella *genome. Click here for file

Additional file 4 ***E. coli *pangenome orthologous cluster group identifier (OCG) to protein to reaction information for the *E. coli *pan-GEM**. This file contains four tables, the first contains all gene additions, the second contains all metabolite additions, and the third contains all reaction additions to iEco1339_MG1655 to construct the *E. coli *pan-GEM (Eco1712_pan). The fourth table contains the final OCG to protein to reaction information for Eco1712_pan. Click here for file

Additional file 5 **Pangenome-scale metabolic model representing the *E. coli *pangenome**. SBML format of iEco1712_pan for distribution and use in other modeling environments. Click here for file

Additional file 6 **Quantitative experimental batch growth data for six *E. coli *and one *Salmonella *strain for aerobic and anaerobic conditions**. This file contains 3 tables, the first contains the biomass data (g/L), the second contains glucose data (g/L), and the third contains the optical density data (600 nm and 1 cm cuvette path length) for experimental batch growth of six *E. coli *and one *Salmonella *strain for aerobic or anaerobic conditions. Values highlighted in yellow reflect the timepoints used to determine lineage-specific glucose uptake rates and strain-specific growth rates. Click here for file

Additional file 7 **Experimental and *in silico *carbon source utilization data**. This file contains two tables, the first contains experimental and *in silico *carbon source utilization data during aerobic conditions, and the second contains experimental and *in silico *carbon source utilization data during anaerobic conditions for six *E. coli *and a *Salmonella *strain. Values highlighted in blue represent false negatives and those highlighted in magenta represent false positives. Click here for file

Additional file 8 **Genome-scale metabolic model representing the ancestral core of *E. coli***. SBML format of iEco1053_core for distribution and use in other modeling environments. Click here for file

Additional file 9 **Necessary orphan reactions required for biomass production**. List of metabolic reactions without corresponding genes necessary for each *E. coli *strain-specific GEM for biomass production in minimal media with glucose added as the sole carbon source. Click here for file

Additional file 10 **Genome-scale metabolic model for *E. coli *K-12 strain W3110**. SBML format of iEco1335_W3110 for distribution and use in other modeling environments. Click here for file

Additional file 11 **Genome-scale metabolic model for enterohemorrhagic *E. coli *O157:H7 strain EDL933**. SBML format of iEco1344_EDL933 for distribution and use in other modeling environments. Click here for file

Additional file 12 **Genome-scale metabolic model for enterohemorrhagic *E. coli *O157:H7 strain Sakai**. SBML format of iEco1345_Sakai for distribution and use in other modeling environments. Click here for file

Additional file 13 **Genome-scale metabolic model for uropathogenic *E. coli *strain CFT073**. SBML format of iEco1288_CFT073 for distribution and use in other modeling environments. Click here for file

Additional file 14 **Genome-scale metabolic model for uropathogenic *E. coli *strain UTI89**. SBML format of iEco1301_UTI89 for distribution and use in other modeling environments. Click here for file

Additional file 15 **Deleted reactions for strain-specific *E. coli *GEMs and the *E. coli *ancestral core GEM**. This file contains three tables; the first contains all gene and corresponding reactions deleted for five *E. coli *strains in comparison to Eco1339_MG1655, the second contains all deleted genes corresponding to isozymes for five E. coli strains in comparison to Eco1339_MG1655, the third contains all reactions deleted from Eco1339_MG1655 to generate the *E. coli *ancestral core GEM (iEco1053_core). Click here for file

Additional file 16 **Reactions corresponding to essential gene predictions for all six strain-specific *E. coli *GEMs and for the *E. coli *ancestral core GEM**. This file contains three tables, the first contains all predicted essential reactions during both aerobic and anaerobic conditions, the second contains all anaerobic-specific predicted essential reactions, the third contains predicted strain- or core-specific essential reactions for all six strain-specific *E. coli *GEMs and for the *E. coli *ancestral core *E. coli *GEM. Click here for file
